# Fractional optimal control of COVID-19 pandemic model with generalized Mittag-Leffler function

**DOI:** 10.1186/s13662-021-03546-y

**Published:** 2021-08-19

**Authors:** Amir Khan, Rahat Zarin, Usa Wannasingha Humphries, Ali Akgül, Anwar Saeed, Taza Gul

**Affiliations:** 1 Department of Mathematics, Faculty of Science, King Mongkut’s University of Technology, Thonburi (KMUTT), 126 Pracha-Uthit Road, Bang Mod, Thrung Khru, Bangkok, 10140 Thailand; 2 Department of Mathematics and Statistics, University of Swat, Khyber Pakhtunkhawa, Pakistan; 3 Department of Basic Sciences, University of Engineering and Technology, Peshawar, Pakistan; 4 Department of Mathematics, Art and Science Faculty of Science, Siirt University, TR-56100 Siirt, Turkey; 5 Center of Excellence in Theoretical and Computational Science (TaCS-CoE), Faculty of Science, King Mongkut’s University of Technology Thonburi (KMUTT), 126 Pracha Uthit Rd., Bang Mod, Thung Khru, Bangkok, 10140 Thailand; 6 Mathematics Department, City University of Science and Information Technology, Peshawar, Pakistan

**Keywords:** Pandemic model, Mittag-Leffler function, Stability analysis, Optimal control, Sensitivity analysis, Numerical simulations

## Abstract

In this paper, we consider a fractional COVID-19 epidemic model with a convex incidence rate. The Atangana–Baleanu fractional operator in the Caputo sense is taken into account. We establish the equilibrium points, basic reproduction number, and local stability at both the equilibrium points. The existence and uniqueness of the solution are proved by using Banach and Leray–Schauder alternative type theorems. For the fractional numerical simulations, we use the Toufik–Atangana scheme. Optimal control analysis is carried out to minimize the infection and maximize the susceptible people.

## Introduction

Corona virus or severe acute respiratory syndrome corona virus 2 (SARS-CoV-2) is a virus that attacks the respiratory system. The virus that causes this disease is called COVID-19 (corona virus disease 2019). The ICTV corona virus disease study group stated that this virus is a species associated with the severe acute respiratory syndrome. COVID-19 was first discovered in humans in December 2019. This outbreak was first detected in Wuhan city, Hubei province, China, in mid-December 2019. The outbreak due to SARS-CoV-2 was declared a global health emergency or pandemic by the World Health Organization (WHO) on January 30, 2020. The Chinese government conducted quarantine in the city of Wuhan on January 23, 2020 as a step to control the pandemic [1].

Modeling in mathematics is a tremendous tool for expressing and dealing with complicated phenomena. Recently, considerable attention has been given to the proposal of mathematical models in comprehending the ailment of infectious nature [2–6]. Many researchers have developed models for the realization and regulation of the outbreak of transmissible diseases in a population. Infectious diseases are the second largest cause of death across the globe. The discipline of infectious diseases will assume added prominence in the twenty-first century in both developed and developing nations. To an unprecedented extent, issues related to infectious diseases in the context of global health are on the agendas of world leaders, health policymakers, and philanthropists. Over the last few years, several researchers have been exploring infectious diseases and their mechanisms using different methods [7–10]. This not only helps to control the spreading of infectious diseases but also aids in everyday life to prevent these diseases. Several researchers have researched epidemic models to examine and monitor various diseases such as avian influenza, hepatitis B, tuberculosis, leishmaniasis [11–13]. Since the existence and annihilation of COVID-19 is subject to numerous parameters of the affected system, we cannot characterize the entire disease system throughout the globe by using a single model. As in the case of COVID-19, the spreading of the disease has a direct relation with the quarantine of the human population. Commonly, we have two types of quarantine: one is susceptible quarantine and the second is infected quarantine. In our work, we take the infected quarantine which means that the people will be quarantined if they are infected.

Fractional calculus is the generalization of classical calculus. To get a better insight into a mathematical model and to deeply understand phenomena, noninteger order operators can be used. Moreover, models involving fractional-order derivatives provide a greater degree of accuracy and are able to abduct the fading memory and spanning behavior. Fractional order differential equation models give more understanding about a disease under consideration [14–17]. Literature has suggested a number of fractional operators with singular and nonsingular kernel [18–21], and their applications can be found in some recent studies [14, 16]. In [22], the authors considered co-dynamics for cancer and hepatitis using a mathematical model with fractional derivative and examined its results. For more details, see [23–25].

We consider the model available in [26] in which the total population is denoted by $N(t)$ and is divided into five groups, namely: susceptible individuals $S(t)$ which denotes individuals vulnerable to the infection; exposed individuals $E(t)$; infectious individuals $I(t)$; quarantined individuals $Q(t)$; and recovered individuals $R(t)$ at time *t*.

1 $$ \textstyle\begin{cases} \frac{d S}{d t} = b- \beta SI(1+\delta I)-(\eta +\mu +d_{3})S(t), \\ \frac{d E}{d t} =\beta SI(1+\delta I)-(\chi +\mu +d_{2})E(t), \\ \frac{d I}{d t} =\lambda E(t)-(\mu +\epsilon +\gamma +d_{1})I(t), \\ \frac{d Q}{d t} =d_{3}S(t)+d_{2}E(t)+d_{1}I(t)-( \mu +\tau )Q(t), \\ \frac{d R}{d t} =\eta S(t)+\tau Q(t)+\gamma I(t)-\mu R(t), \\ S(t)>0, \qquad E(t)\geq {0},\qquad I(t)\geq {0},\qquad Q(t)\geq {0}, \qquad R(t) \geq 0. \end{cases} $$

We reformulated the above model by fractionalizing it with the help of fractional parameter $0 < \chi \leq 1$:

2 $$ \textstyle\begin{cases} {}^{\mathrm{ABC}} \mathbb{D}_{0, t}^{\chi } [S(t) ]= \bar{b}- \bar{\beta } S(t)I(t) (1+\bar{\delta } I(t) ) - (\bar{\eta } + \bar{\mu } + \bar{d}_{3})S(t), \\ {}^{\mathrm{ABC}} \mathbb{D}_{0, t}^{\chi } [E(t) ]= \bar{\beta } S(t) I(t) (1+\bar{\delta } I(t) ) - (\bar{\lambda } + \bar{ \mu } + \bar{d}_{2})E(t), \\ {}^{\mathrm{ABC}} \mathbb{D}_{0, t}^{\chi } [I(t) ] = \bar{\lambda } E(t)-( \bar{\mu } +\bar{\epsilon }+\bar{\gamma } + \bar{d}_{1})I(t), \\ {}^{\mathrm{ABC}} \mathbb{D}_{0, t}^{\chi } [Q(t) ] =\bar{d}_{3}S(t)+ \bar{d}_{2}E(t)+\bar{d}_{1}I(t)-( \bar{\mu } +\bar{\tau })Q(t), \\ {}^{\mathrm{ABC}} \mathbb{D}_{0, t}^{\chi } [R(t) ] = \bar{\eta } S(t)+ \bar{\tau } Q(t)+\bar{\gamma } I(t)-\bar{\mu } R(t), \\ S(t)>0,\qquad E(t)\geq {0},\qquad I(t)\geq {0}, \qquad Q(t)\geq {0},\qquad R(t) \geq 0. \end{cases} $$

The parameters *β̄* and *δ̄* are positive constants, whereas *b̄* is the constant birth rate, *β̄* is the disease transmission coefficient, *μ* is the natural death rate, *ϵ* is the death rate for the disease of infectious individuals, and *λ̄*, *γ̄*, $\bar{d}_{1}$, $\bar{d}_{2}$, $\bar{d}_{3}$, *τ̄* are the state transition rates. *η̄* is the transmission rate from the susceptible to the recovered class which represents those people who have strong immune system.

The organization of our paper is as follows: Sect. 2 deals with the basic definitions which are helpful in the analysis of the coming sections. Also the basic reproduction number as well as equilibrium points are established. The unique positive solution of our proposed model is given. Section 3 is concerned with the local stability of the proposed model. Existence and uniqueness are carried out in Sect. 4. Section 5 deals with the Ulam–Hyers stability of our model. Section 6 depicts some simulations carried out by the Atangana–Toufik scheme. In Sect. 7, optimal control analysis is applied to our model. And in the final Sect. 7, we give the concluding remarks along with the future work.

## Preliminaries

The following definitions of Atangana–Baleanu fractional derivative and integration in the Caputo sense are taken from [27, 28]:

3 $$\begin{aligned}& {}^{AB}_{C} \mathfrak{D}^{\chi }_{0,t} r(t)= \frac{\mathfrak{B}(\chi )}{1-\chi } \int _{0}^{t}E_{\chi } \biggl\{ - \chi \frac{(t-\delta )^{\chi }}{1-\chi } \biggr\} r^{(1)}(\delta ) \,d \delta , \end{aligned}$$

4 $$\begin{aligned}& {}^{{AB}}_{C}I^{\chi }_{0,t}r(t)= \frac{1-\chi }{\mathfrak{B}(\chi )}r(t)+ \frac{\chi }{\mathfrak{B}(\chi )\Gamma (\chi )} \int _{0}^{t} r(\delta ) (t- \delta )^{\chi -1}\,d\delta , \end{aligned}$$

satisfying

5 $$ \bigl\Vert {}^{{AB}}_{C} \mathfrak{D}^{\chi }_{b_{1},t} \bigl(r(t) \bigr) \bigr\Vert < \frac{\mathfrak{B}(\chi )}{1-\chi } \bigl\Vert r(t) \bigr\Vert ,\quad \text{where } \bigl\Vert r(t) \bigr\Vert = \max_{b_{1} \leq t\leq b_{2}} \bigl\vert r(t) \bigr\vert $$

for $r(t) \in C[b_{1},b_{2}]$ and the Lipschitz condition

6 $$ \bigl\Vert {}^{{AB}}_{C} \mathfrak{D}^{\chi }_{b_{1},t}r_{1}(t)-{}^{{AB}}_{C} \mathfrak{D}^{\chi }_{b_{1},t}r_{2}(t) \bigr\Vert < \varpi _{1} \bigl\Vert r_{1}(t)-r_{2}(t) \bigr\Vert . $$

### Basic reproduction number $R_{0}$

The DFE of model (2) is denoted by $E^{0}(S_{0},0,0,Q_{0},R^{0})$, where

$$ S_{0}=\frac{\bar{b}}{\bar{\eta }+\bar{\mu }+\bar{d}_{3}},\qquad Q_{0}= \frac{\bar{d}_{3}S_{0}}{\bar{\mu }+\bar{\tau }}, \qquad R^{0}= \frac{\bar{\eta } S_{0}+\bar{\tau } Q_{0}}{\bar{\mu }}. $$

Similar to the method mentioned in [29], we calculate *F* and *V* as follows:

$$\begin{aligned}& F= \begin{bmatrix} 0 & \bar{\beta } S_{0} \\ 0 & 0 \end{bmatrix},\qquad V= \begin{bmatrix} \bar{\lambda }+\bar{\mu }+\bar{d}_{2} & 0 \\ -\bar{\lambda }& \bar{\mu }+\bar{\epsilon }+\bar{\gamma }+\bar{d}_{1} \end{bmatrix}, \\& V^{-1}= \frac{1}{(\bar{\lambda }+\bar{\mu }+\bar{d}_{2})(\bar{\mu }+\bar{\epsilon }+\bar{\gamma }+\bar{d}_{1})} \begin{bmatrix} \bar{\mu }+\bar{\epsilon }+\bar{\gamma }+\bar{d}_{1} & 0 \\ \bar{\lambda } & \bar{\lambda }+\bar{\mu }+\bar{d}_{2} \end{bmatrix}, \\& FV^{-1}= \begin{bmatrix} \frac{\bar{\beta } S_{0}\bar{\lambda }}{(\bar{\lambda }+\bar{\mu }+\bar{d}_{2})(\bar{\mu }+\bar{\epsilon }+\bar{\gamma }+\bar{d}_{1})} & \frac{\bar{\beta } S_{0}}{\bar{\mu }+\bar{\epsilon }+\bar{\gamma }+\bar{d}_{1}} \\ 0 & 0 \end{bmatrix}. \end{aligned}$$

Hence

$$ R_{0}= \frac{\bar{\lambda } \bar{\beta } \bar{b}}{(\bar{\eta }+\bar{\mu } +\bar{d}_{3})(\bar{\lambda }+\bar{\mu }+\bar{d}_{2})(\bar{\mu } +\bar{\epsilon }+\bar{\gamma }+\bar{d}_{1})}. $$

### Endemic equilibrium point

System (2) is reshaped as

7 $$ \begin{aligned} &S_{*}= \frac{(\bar{\lambda }+\bar{\mu }+\bar{d}_{2})(\bar{\mu }+\bar{\epsilon }+\bar{\gamma } +\bar{d}_{1})I_{*}}{\bar{\lambda } \bar{\beta } I_{*}(1+\bar{\delta } I_{*})} , \\ &E_{*}= \frac{(\bar{\mu }+\bar{\epsilon }+\bar{\gamma }+\bar{d}_{1})I_{*}}{\bar{\lambda }}, \\ &Q_{*}= \frac{\bar{d}_{3}S_{*}+\bar{d}_{2}E_{*}+\bar{d}_{1}I_{*}}{\bar{\mu }+\bar{\tau }}, \\ &R_{*}= \frac{\bar{\eta } S_{*}+\bar{\tau } Q_{*}+\bar{\gamma } I_{*}}{\bar{\mu }}. \end{aligned} $$

Taking into account the above values in

8 $$ \bar{b}-(\bar{\eta }+\bar{\mu }+\bar{d}_{3}) S_{*}-( \bar{\lambda }+ \bar{\mu }+\bar{d}_{2}) E_{*}=0, $$

we get

9 $$ P(I)=A_{1}I^{3}+A_{2}I^{2}+A_{3}I=0, $$

where

10 $$\begin{aligned}& A_{1} = (\bar{\lambda }+\bar{\mu }+\bar{d}_{2}) (\bar{ \mu }+ \bar{\epsilon }+\bar{\gamma }+\bar{d}_{1}) \bar{\beta } \bar{ \delta }, \end{aligned}$$

11 $$\begin{aligned}& A_{2} = - \bigl((\bar{\lambda }+\bar{\mu }+\bar{d}_{2}) (\bar{\mu }+ \bar{\epsilon }+\bar{\gamma }+\bar{d}_{1}) \bar{\beta }+ \bar{b} \bar{\lambda } \bar{\beta } \bar{\delta } \bigr), \end{aligned}$$

12 $$\begin{aligned}& A_{3} = b\bar{\lambda } \bar{\beta }-(\bar{\lambda }+\bar{\mu }+ \bar{d}_{2}) ( \bar{\mu }+\bar{\epsilon }+\bar{\gamma }+ \bar{d}_{1}) (\bar{\eta }+ \bar{\mu }+\bar{d}_{3}). \end{aligned}$$

By Descarte’s rule of the sign if $A_{3}>0$, then (9) has one positive, one negative, and one zero root, and $A_{3}>0$ implies that $R_{0} > 1$. Hence, for $R_{0} > 1$, a unique positive equilibrium exists for the model.

## Local stability

We establish the local stability of system (2) in this section at COVID-19 free point $E^{0}$ as well as at COVID-19 present equilibrium point $E^{*}$.

### Theorem 1

*The COVID*-19 *free equilibrium* (*CFE*) *point* $E^{0}$ *of the proposed fractional order* *SEQIR* *pandemic model* (2) *is locally asymptotically stable if* $R_{0} < 1$.

### Proof

The Jacobian matrix of system (2) at $E^{0}$ is

13 $$ J \bigl(E^{0} \bigr)= \begin{pmatrix} -(\bar{\eta }+\bar{\mu }+\bar{d}_{3}) & 0 & - \frac{\bar{\beta } \bar{b}}{\bar{\eta }+\bar{\mu }+\bar{d}_{3}} & 0 & 0 \\ 0 & -(\bar{\lambda }+\bar{\mu }+\bar{d}_{2}) & \frac{\bar{\beta } \bar{b}}{\bar{\eta }+\bar{\mu }+\bar{d}_{3}} & 0 & 0 \\ 0 & \bar{\lambda } & -(\bar{\mu }+\bar{\epsilon }+\bar{\gamma }+\bar{d}_{1}) & 0 & 0 \\ \bar{d}_{3} & \bar{d}_{2} & \bar{d}_{1} &-(\bar{\mu }+\bar{\tau }) & 0 \\ \bar{\eta } & 0 & \bar{\gamma } & \bar{\tau } & -\bar{\mu } \end{pmatrix}. $$

Therefore, by the Routh–Hurwitz stability conditions for fractional order systems [30], the necessary and sufficient condition is

14 $$ \bigl\vert \arg \bigl(\operatorname{eig}(J) \bigr) \bigr\vert = \bigl\vert \arg (\omega _{i} ) \bigr\vert >\kappa \frac{\pi }{2} $$

for various fractional order models. Therefore, the disease-free equilibrium of system (2) is asymptotically stable if all of the eigenvalues $\omega _{i}$, $i=1,2,3,4,5$, of $J(E^{0})$ satisfy condition (14). Hence, a sufficient condition for the local asymptotic stability of the equilibrium points is that the eigenvalues $\omega _{i}$, $i=1,2,3,4,5$, of the Jacobian matrix $J(E^{0})$ satisfy the condition $\vert \arg (\omega _{i} ) \vert >\kappa \frac{\pi }{2}$. This confirms that fractional order differential equations are, at least, as stable as their integer order counterparts.

The characteristic equation of $J(E^{0})$ is

$$ \bigl(\omega _{1}+(\bar{\eta }+\bar{\mu }+\bar{d}_{3}) \bigr) \bigl(\omega _{2}+( \bar{\mu }+\bar{\tau }) \bigr) (\omega _{3}+\bar{\mu }) \bigl(A\omega ^{2} + B \omega +C \bigr)=0, $$

where

15 $$\begin{aligned}& \begin{aligned} &A= 1, \\ &B= 2\bar{\mu }+\bar{\lambda }+\bar{\epsilon }+\bar{\gamma }+ \bar{d}_{1}+ \bar{d}_{2}, \\ &C= (\bar{\lambda }+\bar{\mu }+\bar{d}_{2}) (\bar{\mu }+\bar{ \epsilon }+ \bar{\gamma }+\bar{d}_{1})- \frac{\bar{\beta }\bar{\lambda } b}{\bar{\eta }+\bar{\mu }+\bar{d}_{3}}, \end{aligned} \\& (\bar{\lambda }+\bar{\mu }+\bar{d}_{2}) (\bar{\mu }+\bar{\epsilon }+ \bar{\gamma }+\bar{d}_{1})- \frac{\bar{\beta }\bar{\lambda } b}{\bar{\eta }+\bar{\mu }+\bar{d}_{3}}>0, \\& (\bar{\lambda }+\bar{\mu }+\bar{d}_{2}) (\bar{\mu }+\bar{\epsilon }+ \bar{\gamma }+\bar{d}_{1}) (1-R_{0})>0. \end{aligned}$$

This shows that, for $R_{0} < 1$, the quadratic equation $(A\omega ^{2} + B \omega +C)=0$ has all terms positive, and thus its roots must all be negative. Meanings $\lambda _{4,5}<0$, all of the eigenvalues $\omega _{i}$ for $i=1,2,3,4,5$, satisfy the condition given by (14). Therefore, all the eigenvalues have negative real parts if $R_{0} < 1$. This completes the proof. □

### At pandemic equilibrium point

#### Lemma 1

*LetMbe a*$3\times 3$*real matrix*. *If*$\operatorname{tr}(M)$, $\det (M)$, *and*$\det M^{[2]}$*are all negative*, *then all eigenvalues ofMhave negative real parts*.

#### Theorem 2

*If*$R_{0}>1$, *then the pandemic equilibrium*$E^{*}$*of the proposed fractional orderSEQIRpandemic model* (2) *is locally asymptotically stable*.

#### Proof

The Jacobian matrix of system (2) at $E^{*}$ is

$$\begin{aligned} J^{| *| }= \begin{pmatrix} -a_{11} & 0 & - \bar{\beta } S_{*}(1+2\bar{\delta } I_{*}) & 0 & 0 \\ \bar{\beta } I_{*}(1+\bar{\delta } I_{*} ) & -(\bar{\lambda }+\bar{\mu }+ \bar{d}_{2}) & \bar{\beta } S_{*}(1+2\bar{\delta } I_{*}) & 0 & 0 \\ 0 & \bar{\lambda } & -(\bar{\mu }+\bar{\epsilon }+\bar{\gamma }+\bar{d}_{1}) & 0 & 0 \\ \bar{d}_{3} & \bar{d}_{2} & \bar{d}_{1} &-(\bar{\mu }+\bar{\tau }) & 0 \\ \bar{\eta } & 0 & \bar{\gamma } & \bar{\tau } & -\bar{\mu } \end{pmatrix}, \end{aligned}$$

where $a_{11}=(\bar{\beta } I_{*}(1+\bar{\delta } I_{*})+(\bar{\eta }+ \bar{\mu }+\bar{d}_{3}))$.

Therefore, by the Routh–Hurwitz stability conditions for fractional order systems [30], the necessary and sufficient condition is

16 $$ \bigl\vert \arg \bigl(\operatorname{eig} \bigl(J^{| *| } \bigr) \bigr) \bigr\vert = \bigl\vert \arg ( \omega _{i} ) \bigr\vert >\kappa \frac{\pi }{2} $$

for various fractional order models. Therefore, the disease-free equilibrium of system (2) is asymptotically stable if all of the eigenvalues $\omega _{i}$, $i=1,2,3,4,5$, of $J^{| *| }(E^{*})$ satisfy condition (16). Hence, a sufficient condition for the local asymptotic stability of the equilibrium points is that the eigenvalues $\omega _{i}$, $i=1,2,3,4,5$, of the Jacobian matrix $J^{| *| }(E^{*})$ satisfy the condition $\vert \arg (\omega _{i} ) \vert >\kappa \frac{\pi }{2}$. This confirms that fractional order differential equations are, at least, as stable as their integer order counterparts.

Here, $\omega _{1} = -\bar{\mu }$, $\omega _{2} = -(\bar{\mu }+\bar{\tau })$, and we consider the following matrix for the rest of eigenvalues:

$$ J^{| *| }_{1}= \begin{pmatrix} -(\bar{\beta } I_{*}(1+\bar{\delta } I_{*})+(\bar{\eta }+\bar{\mu }+ \bar{d}_{3})) & 0 & - \bar{\beta } S_{*}(1+2\bar{\delta } I_{*}) \\ \bar{\beta } I_{*}(1+\bar{\delta } I_{*} ) & -(\bar{\lambda }+\bar{\mu }+ \bar{d}_{2}) & \bar{\beta } S_{*}(1+2\bar{\delta } I_{*}) \\ 0 & \bar{\lambda } & -(\bar{\mu }+\bar{\epsilon }+\bar{\gamma }+\bar{d}_{1}) \end{pmatrix}. $$

From the Jacobian matrix $J^{| *| }_{1}$ we have

$$ \operatorname{tr} \bigl(J^{| *| }_{1} \bigr)=- \bigl[ \bigl( \bar{\beta } I_{*}(1+\bar{\delta } I_{*})+( \bar{ \eta }+ \bar{\mu }+\bar{d}_{3}) \bigr)+(\bar{\lambda }+\bar{\mu }+ \bar{d}_{2})+( \bar{\mu }+\bar{\epsilon }+\bar{\gamma }+ \bar{d}_{1}) \bigr]< 0, $$

also

$$\begin{aligned}& \begin{aligned} \det \bigl(J^{| *| }_{1} \bigr)={}&{-} \bigl[ \bigl(\bar{\beta } I_{*}(1+ \bar{\delta } I_{*})+( \bar{\eta }+\bar{\mu }+\bar{d}_{3}) \bigr) \bigl(( \bar{\lambda }+ \bar{\mu }+\bar{d}_{2}) (\bar{\mu }+\bar{ \epsilon }+ \bar{\gamma }+ \bar{d}_{1}) \\ &{}-\bar{\lambda } \bar{\beta } S_{*}(1+2 \bar{ \delta } I_{*}) + \bar{\lambda } \bar{\beta } S_{*}(1+2\bar{\delta } I_{*}) \bar{\beta } S_{*} I_{*}(1+2\bar{ \delta } I_{*}) \bigr) \bigr], \\ ={}&{-} \bigl[ \bigl(\bar{\beta } I_{*}(1+\bar{\delta } I_{*})+(\bar{\eta }+\bar{\mu }+ \bar{d}_{3}) \bigr) \bigl((\bar{\lambda }+\bar{\mu }+\bar{d}_{2}) (\bar{\mu }+ \bar{ \epsilon }+\bar{\gamma }+\bar{d}_{1}) \\ &{}+\bar{\lambda } \bar{\beta } S_{*}(1+2\bar{\delta } I_{*}) \bigl[\bar{\beta } S_{*} I_{*}(1+2 \bar{\delta } I_{*})-1 \bigr] \bigr) \bigr], \end{aligned} \\& \begin{aligned} \det \bigl(J^{| *| }_{1} \bigr)={}&{-} \bigl[ \bigl(\bar{\beta } I_{*}(1+ \bar{\delta } I_{*})+( \bar{\eta }+\bar{\mu }+\bar{d}_{3}) \bigr) \bigl(( \bar{\lambda }+ \bar{\mu }+\bar{d}_{2}) (\bar{\mu }+\bar{ \epsilon }+ \bar{\gamma }+ \bar{d}_{1}) \\ &{}+\bar{\lambda } \bar{\beta } S_{*}(1+2\bar{\delta } I_{*}) \bigl[\bar{\beta } S_{*} I_{*}(1+2 \bar{\delta } I_{*})-1 \bigr] \bigr) \bigr]< 0. \end{aligned} \end{aligned}$$

Further, the second additive compound matrix is

$$ J^{[2]}= \begin{pmatrix} -\bar{\beta } I(1+\bar{\delta } I)-m & \bar{\beta } S(1+2\bar{\delta } I) &\bar{\beta } S(1+2\bar{\delta } I) \\ \bar{\lambda } & -\bar{\beta } I(1+\bar{\delta } I)-n & 0 \\ 0 & \bar{\beta } I(1+\bar{\delta } I) &-k \end{pmatrix}, $$

where

$$ \begin{aligned} & m =2\bar{\mu }+\bar{\eta }+\bar{\lambda }+ \bar{d}_{2}+\bar{d}_{3}, \\ & n =2\bar{\mu }+\bar{\eta }+\varepsilon ^{\chi }+\bar{\gamma }+ \bar{d}_{1}+ \bar{d}_{3}, \\ & k=2\bar{\mu }+\bar{\lambda }+\varepsilon ^{\chi }+\bar{\gamma }+ \bar{d}_{1}+\bar{d}_{2}. \end{aligned} $$

Hence

$$ \begin{aligned} \det \bigl(J^{[2]} \bigr)={}&{-} \bigl[ \bigl( \bar{\beta } I_{*}(1+\bar{\delta } I_{*})+m \bigr) \bigl( \bar{\beta } I_{*}(1+\bar{\delta } I_{*})+n \bigr)k+ \bigl(\bar{\beta } I_{*}(1+ \bar{\delta } I_{*}) \bigr) \bigl(\bar{\lambda } \bar{\beta } I_{*}(1+ \bar{\delta } I_{*}) \bigr) \\ &{}- \bigl(\bar{\beta } S_{*}(1+\bar{\delta } 2 \bar{\delta } I_{*}) \bar{\lambda } k \bigr) \bigr]< 0. \end{aligned} $$

Therefore, by Lemma 1, all of the eigenvalues $\omega _{i}$ for $i=1,2,3,4,5$ satisfy the condition given by (16). Thus, the pandemic equilibrium point $E^{*}$ is locally asymptotically stable. □

## Existence and uniqueness

We denote a Banach space by $D(W)$ with $W =[0,b]$ containing a real-valued continuous function with sup norm and $P=D(W) \times D(W) \times D(W) \times D(W) \times D(W) $ with norm $\Vert (S, E, I, Q, R ) \Vert =\|S\|+\|E\|+ \Vert I \Vert + \Vert Q \Vert +\|R\|$, where $\|S\|=\sup_{t \in J}|S(t)|$, $\|E\|=\sup_{t \in j}|E(t)|$, $\Vert I \Vert =\sup_{t \in j}|I(t)|$, $\Vert Q \Vert =\sup_{t \in j}|Q(t)|$, $\Vert R \Vert =\sup_{t \in j}|R(t)|$. By using the ABC integral operator on model (2), we get

17 $$ \textstyle\begin{cases} S(t)-S(0)={}^{\mathrm{ABC}} \mathbb{D}_{0, t}^{\chi } [S(t) ] \{ \bar{b}- \bar{\beta } S(t)I(t) (1+\bar{\delta } I(t) ) - ( \bar{\eta } + \bar{\mu } + \bar{d}_{3})S(t) \} , \\ E(t)-E(0)={}^{\mathrm{ABC}} \mathbb{D}_{0, t}^{\chi } [E(t) ] \{ \bar{\beta } S(t) I(t) (1+\bar{\delta } I(t) ) - (\bar{\lambda } + \bar{\mu } + \bar{d}_{2})E(t) \} , \\ I(t)-Q(0)={}^{\mathrm{ABC}} \mathbb{D}_{0, t}^{\chi } [I(t) ] \{ \bar{\lambda } E(t)-(\bar{\mu } +\bar{\epsilon }+\bar{ \gamma } +\bar{d}_{1})I(t) \} , \\ Q(t)-I(0)={}^{\mathrm{ABC}} \mathbb{D}_{0, t}^{\chi } [Q(t) ] \{ \bar{d}_{3}S(t)+\bar{d}_{2}E(t)+ \bar{d}_{1}I(t)-(\bar{\mu } + \bar{\tau })Q(t) \} , \\ R(t)-R(0)={}^{\mathrm{ABC}} \mathbb{D}_{0, t}^{\chi } [R(t) ] \{ \bar{\eta } S(t)+\bar{\tau } Q(t)+\bar{\gamma } I(t)- \bar{\mu } R(t) \} . \end{cases} $$

Now, using equation (3), we obtain

18 $$ \begin{aligned} &S(t)-S(0)= \frac{1-\chi }{B(\chi )} \mathfrak{K}_{1} \bigl(\chi , t, S(t) \bigr)+ \frac{\chi }{B(\chi ) \Gamma (\chi )} \times \int _{0}^{t}(t- \vartheta )^{\chi -1} \mathfrak{K}_{1} \bigl(\chi , \vartheta , S( \vartheta ) \bigr) \,d\vartheta , \\ &E(t)-E(0)= \frac{1-\chi }{B(\chi )} \mathfrak{K}_{2} \bigl(\chi , t, E(t) \bigr)+ \frac{\chi }{B(\chi ) \Gamma (\chi )} \times \int _{0}^{t}(t- \vartheta )^{\chi -1} \mathfrak{K}_{2} \bigl(\chi , \vartheta , E( \vartheta ) \bigr) \,d\vartheta , \\ &I(t)-I(0)= \frac{1-\chi }{B(\chi )} \mathfrak{K}_{3} \bigl(\chi , t, I(t) \bigr)+ \frac{\chi }{B(\chi ) \Gamma (\chi )} \times \int _{0}^{t}(t- \vartheta )^{\chi -1} \mathfrak{K}_{3} \bigl(\chi , \vartheta , Q( \vartheta ) \bigr) \,d\vartheta , \\ &Q(t)-Q(0)= \frac{1-\chi }{B(\chi )} \mathfrak{K}_{4} \bigl(\chi , t, Q(t) \bigr)+ \frac{\chi }{B(\chi ) \Gamma (\chi )} \times \int _{0}^{t}(t- \vartheta )^{\chi -1} \mathfrak{K}_{4} \bigl(\chi , \vartheta , I( \vartheta ) \bigr) \,d\vartheta , \\ &R(t)-R(0)= \frac{1-\chi }{B(\chi )} \mathfrak{K}_{5} \bigl(\chi , t, R(t) \bigr)+ \frac{\chi }{B(\chi ) \Gamma (\chi )} \times \int _{0}^{t}(t- \vartheta )^{\chi -1} \mathfrak{K}_{5} \bigl(\chi , \vartheta , R( \vartheta ) \bigr) \,d\vartheta , \end{aligned} $$

where

19 $$\begin{aligned}& \mathfrak{K}_{1} \bigl( \chi , t, S(t) \bigr)= \bar{b}- \bar{\beta } S(t)I(t) \bigl(1+ \bar{\delta } I(t) \bigr) - (\bar{\eta } + \bar{\mu } + \bar{d}_{3})S(t), \\& \mathfrak{K}_{2} \bigl(\chi , t, E(t) \bigr)=\bar{\beta } S(t) I(t) \bigl(1+ \bar{\delta } I(t) \bigr) - (\bar{\lambda } + \bar{\mu } + \bar{d}_{2})E(t), \\& \mathfrak{K}_{3} \bigl(\chi , t, I(t) \bigr)=\chi {E(t)} - (\bar{ \mu } + \bar{\epsilon } + \bar{\gamma } + \bar{d}_{1})I(t), \\& \mathfrak{K}_{4} \bigl(\chi , t, Q(t) \bigr)=\bar{d}_{3}S(t) + \bar{d}_{2}E(t) +\bar{d}_{1}I(t) - (\bar{\mu } + \bar{\tau })Q(t) , \\& \mathfrak{K}_{5} \bigl(\chi , t, R(t) \bigr)=\bar{\eta } {S(t)} + \bar{\tau } {Q(t)} + \bar{\gamma } {I(t)} - \bar{\mu } {R(t)}. \end{aligned}$$

The $\mathfrak{K}_{1}$, $\mathfrak{K}_{2}$, $\mathfrak{K}_{3}$, $\mathfrak{K}_{4}$, and $\mathfrak{K}_{5} $ satisfy the Lipschitz condition only if $S(t)$, $E(t)$, $I(t)$, $Q(t)$, and $R(t)$ possess an upper bound. Supposing $S(t)$ and $S^{*}(t)$ are couple functions, we have

20 $$ \bigl\Vert \mathfrak{K}_{1} \bigl(\chi , t, S(t) \bigr)- \mathfrak{K}_{1} \bigl(\chi , t, S^{*}(t) \bigr) \bigr\Vert =\bigl\| - \bigl(\bar{\beta } I (1+ \bar{\delta } I) + \bar{\eta } +\bar{\mu } + \bar{d}_{3} \bigr) \bigl(S(t)-S^{*}(t) \bigr)\bigr\| . $$

Considering

$$ \eta _{1}:=\bigl\| - \bigl( \bar{\beta } I (1+\bar{\delta } I) + \bar{ \eta } +\bar{\mu } + \bar{d}_{3} \bigr)\bigr\| , $$

we get

21 $$ \bigl\Vert \mathfrak{K}_{1} \bigl(\chi , t, S(t) \bigr)-\mathfrak{K}_{1} \bigl(\chi , t, S^{*}(t) \bigr) \bigr\Vert \leq \eta _{1} \bigl\Vert S(t)-S^{*}(t) \bigr\Vert . $$

Similarly,

22 $$ \begin{aligned} & \bigl\Vert \mathfrak{K}_{2} \bigl(\chi , t, E(t) \bigr)-\mathfrak{K}_{2} \bigl(\chi , t, E^{*}(t) \bigr) \bigr\Vert \leq \eta _{2} \bigl\Vert E(t)-E^{*}(t) \bigr\Vert , \\ & \bigl\Vert \mathfrak{K}_{3} \bigl(\chi , t, I(t) \bigr)- \mathfrak{K}_{3} \bigl(\chi , t, I^{*}(t) \bigr) \bigr\Vert \leq \eta _{3} \bigl\Vert I(t)-I^{*}(t) \bigr\Vert , \\ & \bigl\Vert \mathfrak{K}_{4} \bigl(\chi , t, Q(t) \bigr)- \mathfrak{K}_{4} \bigl(\chi , t, Q^{*}(t) \bigr) \bigr\Vert \leq \eta _{4} \bigl\Vert Q(t)-Q^{*}(t) \bigr\Vert , \\ & \bigl\Vert \mathfrak{K}_{5} \bigl(\chi , t, R(t) \bigr)- \mathfrak{K}_{5} \bigl(\chi , t, R^{*}(t) \bigr) \bigr\Vert \leq \eta _{5} \bigl\Vert R(t)-R^{*}(t) \bigr\Vert , \end{aligned} $$

where

$$ \begin{aligned} &\eta _{2}= \bigl\Vert - (\bar{\lambda } + \bar{\mu } + \bar{d}_{2} ) \bigr\Vert \\ &\eta _{3}= \bigl\Vert - (\bar{\mu } + \bar{\epsilon } + \bar{ \gamma } + \bar{d}_{1} ) \bigr\Vert \\ &\eta _{4}= \bigl\Vert - (\bar{\mu } + \bar{\tau } ) \bigr\Vert \\ &\eta _{5}= \bigl\Vert - (\bar{\mu } ) \bigr\Vert , \end{aligned} $$

which shows that the Lipschitz condition holds. Continuing in a recursive manner, (18) gives us

23 $$\begin{aligned} &S_{n}(t)-S(0)= \frac{1-\chi }{B(\chi )} \mathfrak{K}_{1} \bigl(\chi , t, S_{n-1}(t) \bigr) \\ &\hphantom{S_{n}(t)-S(0)={}}{}+ \frac{\chi }{B(\chi ) \Gamma (\chi )} \times \int _{0}^{t}(t- \vartheta )^{\chi -1} \mathfrak{K}_{1} \bigl(\chi , \vartheta , S_{n-1}( \vartheta ) \bigr) \,d\vartheta , \\ &E_{n}(t)-E(0)= \frac{1-\chi }{B(\chi )} \mathfrak{K}_{2} \bigl(\chi , t, E_{n-1}(t) \bigr) \\ &\hphantom{E_{n}(t)-E(0)={}}{}+ \frac{\chi }{B(\chi ) \Gamma (\chi )} \times \int _{0}^{t}(t- \vartheta )^{\chi -1} \mathfrak{K}_{2} \bigl(\chi , \vartheta , E_{n-1}( \vartheta ) \bigr) \,d\vartheta , \\ &I_{n}(t)-I(0)= \frac{1-\chi }{B(\chi )} \mathfrak{K}_{3} \bigl(\chi , t, I_{n-1}(t) \bigr) \\ &\hphantom{I_{n}(t)-I(0)={}}{}+ \frac{\chi }{B(\chi ) \Gamma (\chi )} \times \int _{0}^{t}(t- \vartheta )^{\chi -1} \mathfrak{K}_{3} \bigl(\chi , \vartheta , I_{n-1}( \vartheta ) \bigr) \,d\vartheta , \\ &Q_{n}(t)-Q(0)= \frac{1-\chi }{B(\chi )} \mathfrak{K}_{4} \bigl(\chi , t, Q_{n-1}(t) \bigr) \\ &\hphantom{Q_{n}(t)-Q(0)={}}{}+ \frac{\chi }{B(\chi ) \Gamma (\chi )} \times \int _{0}^{t}(t- \vartheta )^{\chi -1} \mathfrak{K}_{4} \bigl(\chi , \vartheta , Q_{n-1}( \vartheta ) \bigr) \,d\vartheta , \\ &R_{n}(t)-R(0)= \frac{1-\chi }{B(\chi )} \mathfrak{K}_{5} \bigl(\chi , t, R_{n-1}(t) \bigr) \\ &\hphantom{R_{n}(t)-R(0)={}}{}+ \frac{\chi }{B(\chi ) \Gamma (\chi )} \times \int _{0}^{t}(t- \vartheta )^{\chi -1} \mathfrak{K}_{5} \bigl(\chi , \vartheta , R_{n-1}( \vartheta ) \bigr) \,d\vartheta , \end{aligned}$$

together with $S_{0}(t)=S(0)$, $E_{0}(t)=E(0)$, $I_{0}(t)=I(0)$, $Q_{0}(t)=Q(0)$, and $R_{0}(t)=R(0)$. Difference of consecutive terms yields

24 $$ \begin{aligned} &\begin{aligned} \Xi _{S, n}(t)={}& S_{n}(t)-S_{n-1}(t)=\frac{1-\chi }{B(\chi )} \bigl( \mathfrak{K}_{1} \bigl(\chi , t, S_{n-1}(t) \bigr)- \mathfrak{K}_{1} \bigl(\chi , t, S_{n-2}(t) \bigr) \bigr) \\ &{}+\frac{\chi }{B(\chi ) \Gamma (\chi )} \int _{0}^{t}(t-\vartheta )^{ \chi -1} \bigl(\mathfrak{K}_{1} \bigl(\chi , \vartheta , S_{n-1}( \vartheta ) \bigr)-\mathfrak{K}_{1} \bigl(\chi , \vartheta , S_{n-2}(\vartheta ) \bigr) \bigr) \,d\vartheta, \end{aligned} \\ &\begin{aligned} \Xi _{E, n}(t)={}& E_{n}(t)-E_{n-1}(t)= \frac{1-\chi }{B(\chi )} \bigl( \mathfrak{K}_{2} \bigl(\chi , t, E_{n-1}(t) \bigr)-\mathfrak{K}_{2} \bigl(\chi , t, E_{n-2}(t) \bigr) \bigr) \\ &{}+\frac{\chi }{B(\chi ) \Gamma (\chi )} \int _{0}^{l}(t-\vartheta )^{ \chi -1} \bigl(\mathfrak{K}_{2} \bigl(\chi , \vartheta , E_{n-1}( \vartheta ) \bigr)-\mathfrak{K}_{2} \bigl(\chi , \vartheta , E_{n-2}(\vartheta ) \bigr) \bigr) \,d\vartheta, \end{aligned} \\ &\begin{aligned} \Xi _{I, n}(t)={}& I_{1 n}(t)-I_{n-1}(t) = \frac{1-\chi }{B(\chi )} \bigl( \mathfrak{K}_{3} \bigl(\chi , t, I_{n-1}(t) \bigr)-\mathfrak{K}_{3} \bigl(\chi , t, I_{n-2}(t) \bigr) \bigr) \\ &{}+\frac{\chi }{B(\chi ) \Gamma (\chi )} \int _{0}^{t}(t-\vartheta )^{ \chi -1} \bigl(\mathfrak{K}_{3} \bigl(\chi , \vartheta , I_{n-1}( \vartheta ) \bigr)-\mathfrak{K}_{3} \bigl(\chi , \vartheta , I_{n-2}(\vartheta ) \bigr) \bigr) \,d\vartheta, \end{aligned} \\ &\begin{aligned} \Xi _{Q, n}(t)={}& Q_{2 n}(t)-Q_{n-1}(t) = \frac{1-\chi }{B(\chi )} \bigl( \mathfrak{K}_{4} \bigl(\chi , t, Q_{n-1}(t) \bigr)-\mathfrak{K}_{4} \bigl(\chi , t, Q_{n-2}(t) \bigr) \bigr) \\ &{}+\frac{\chi }{B(\chi ) \Gamma (\chi )} \int _{0}^{t}(t-\vartheta )^{ \chi -1} \bigl(\mathfrak{K}_{4} \bigl(\chi , \vartheta , Q_{n-1}( \vartheta ) \bigr)-\mathfrak{K}_{4} \bigl(\chi , \vartheta , Q_{n-2}(\vartheta ) \bigr) \bigr) \,d\vartheta, \end{aligned} \\ &\begin{aligned} \Xi _{R, n}(t)={}& F_{n}(t)-R_{n-1}(t)= \frac{1-\chi }{B(\chi )} \bigl( \mathfrak{K}_{5} \bigl(\chi , t, R_{n-1}(t) \bigr)-\mathfrak{K}_{5} \bigl(\chi , t, R_{n-2}(t) \bigr) \bigr) \\ &{}+\frac{\chi }{B(\chi ) \Gamma (\chi )} \int _{0}^{t}(t-\vartheta )^{ \chi -1} \bigl(\mathfrak{K}_{5} \bigl(\chi , \vartheta , R_{n-1}( \vartheta ) \bigr)-\mathfrak{K}_{5} \bigl(\chi , \vartheta , R_{n-2}(\vartheta ) \bigr) \bigr) \,d\vartheta . \end{aligned} \end{aligned} $$

Noting that

25 $$ \begin{aligned} &S_{n}(t)=\sum _{i=0}^{n} \Xi _{S, i}(t),\qquad E_{n}(t)= \sum_{i=0}^{n} \Xi _{E, i}(t),\qquad I_{n}(t)=\sum_{i=0}^{n} \Xi _{I, i}(t), \\ &Q_{n}(t)=\sum_{i=0}^{n} \Xi _{Q, i}(t),\qquad R_{n}(t)=\sum_{i=0}^{n} \Xi _{R, i}(t). \end{aligned} $$

Taking into account Eqs. (21)–(22) and considering that

26 $$\begin{aligned} &\Xi _{S, n-1}(t)=S_{n-1}(t)-S_{n-2}(t),\qquad \Xi _{E, n-1}(t)=E_{n-1}(t)-E_{n-2}(t), \\ &\Xi _{I, n-1}(t)=I_{n-1}(t)-I_{n-2}(t),\qquad \Xi _{Q, n-1}(t)=Q_{n-1}(t)-Q_{n-2}(t), \\ &\Xi _{R, n-1}(t)=R_{n-1}(t)-R_{n-2}(t), \end{aligned}$$

we reach

27 $$ \begin{aligned} & \bigl\Vert \Xi _{S, n}(t) \bigr\Vert \leq \frac{1-\chi }{B(\chi )} \eta _{1} \bigl\Vert \Xi _{S, n-1}(t) \bigr\Vert \frac{\chi }{B(\chi ) \Gamma (\chi )} \eta _{1} \times \int _{0}^{t}(t- \vartheta )^{\chi -1} \bigl\Vert \Xi _{S, n-1}(\vartheta ) \bigr\Vert \,d\vartheta, \\ & \bigl\Vert \Xi _{E, n}(t) \bigr\Vert \leq \frac{1-\chi }{B(\chi )} \eta _{2} \bigl\Vert \Xi _{E, n-1}(t) \bigr\Vert \frac{\chi }{B(\chi ) \Gamma (\chi )} \eta _{2} \times \int _{0}^{t}(t- \vartheta )^{\chi -1} \bigl\Vert \Xi _{E, n-1}(\vartheta ) \bigr\Vert \,d\vartheta, \\ & \bigl\Vert \Xi _{I, n}(t) \bigr\Vert \leq \frac{1-\chi }{B(\chi )} \eta _{3} \bigl\Vert \Xi _{I, n-1}(t) \bigr\Vert \frac{\chi }{B(\chi ) \Gamma (\chi )} \eta _{3} \times \int _{0}^{t}(t- \vartheta )^{\chi -1} \bigl\Vert \Xi _{I, n-1}(\vartheta ) \bigr\Vert \,d\vartheta, \\ & \bigl\Vert \Xi _{Q, n}(t) \bigr\Vert \leq \frac{1-\chi }{B(\chi )} \eta _{4} \bigl\Vert \Xi _{Q, n-1}(t) \bigr\Vert \frac{\chi }{B(\chi ) \Gamma (\chi )} \eta _{4} \times \int _{0}^{t}(t- \vartheta )^{\chi -1} \bigl\Vert \Xi _{Q, n-1}(\vartheta ) \bigr\Vert \,d\vartheta, \\ & \bigl\Vert \Xi _{R, n}(t) \bigr\Vert \leq \frac{1-\chi }{B(\chi )} \eta _{5} \bigl\Vert \Xi _{R, n-1}(t) \bigr\Vert \frac{\chi }{B(\chi ) \Gamma (\chi )} \eta _{5} \times \int _{0}^{t}(t- \vartheta )^{\chi -1} \bigl\Vert \Xi _{R, n-1}(\vartheta ) \bigr\Vert \,d\vartheta . \end{aligned} $$

### Theorem 3

*System* (2) *has a unique solution for* $t \in [0,b]$ *subject to the condition if*

28 $$ \frac{1-\chi }{B(\chi )} \eta _{i} + \frac{\chi }{B(\chi ) \Gamma (\chi )} \bar{b} \eta _{i}< 1, \quad i=1,2, \ldots , 5, $$

*holds*.

### Proof

Since $S(t)$, $E(t)$, $I(t)$, $Q(t)$, and $R(t)$ are bounded functions and Eqs. (21)–(22) hold, in a recursive manner Eq. (27) leads to

29 $$ \begin{aligned} & \bigl\Vert \Xi _{S, n}(t) \bigr\Vert \leq \bigl\Vert S_{0}(t) \bigr\Vert \biggl( \frac{1-\chi }{B(\chi )} \eta _{1} + \frac{\chi \bar{b}}{B(\chi ) \Gamma (\chi )} \eta _{1} \biggr)^{n}, \\ & \bigl\Vert \Xi _{E, n}(t) \bigr\Vert \leq \bigl\Vert E_{0}(t) \bigr\Vert \biggl( \frac{1-\chi }{B(\chi )} \eta _{3} + \frac{\chi \bar{b}}{B(\chi ) \Gamma (\chi )} \eta _{2} \biggr)^{n}, \\ & \bigl\Vert \Xi _{I, n}(t) \bigr\Vert \leq \bigl\Vert I_{0}(t) \bigr\Vert \biggl( \frac{1-\chi }{B(\chi )} \eta _{3} + \frac{\chi \bar{b}}{B(\chi ) \Gamma (\chi )} \eta _{3} \biggr)^{n}, \\ & \bigl\Vert \Xi _{Q, n}(t) \bigr\Vert \leq \bigl\Vert Q_{0}(t) \bigr\Vert \biggl( \frac{1-\chi }{B(\chi )} \eta _{4} + \frac{\chi \bar{b}}{B(\chi ) \Gamma (\chi )} \eta _{4} \biggr)^{n}, \\ & \bigl\Vert \Xi _{R, n}(t) \bigr\Vert \leq \bigl\Vert R_{0}(t) \bigr\Vert \biggl( \frac{1-\chi }{B(\chi )} \eta _{5} + \frac{\chi \bar{b}}{B(\chi ) \Gamma (\chi )} \eta _{5} \biggr)^{n}. \end{aligned} $$

So

$$\begin{aligned}& \bigl\Vert \Xi _{S, n}(t) \bigr\Vert \rightarrow 0,\qquad \bigl\Vert \Xi _{E, n}(t) \bigr\Vert \rightarrow 0, \qquad \bigl\Vert \Xi _{I, n}(t) \bigr\Vert \rightarrow 0, \\& \bigl\Vert \Xi _{Q, n}(t) \bigr\Vert \rightarrow 0,\qquad \bigl\Vert \Xi _{R, n}(t) \bigr\Vert \rightarrow 0 \end{aligned}$$

as $n \rightarrow \infty $. Incorporating the triangle inequality, and for any *k*, Eq. (29) yields

30 $$ \begin{aligned} & \bigl\Vert S_{n+k}(t)-S_{n}(t) \bigr\Vert \leq \sum_{j=n+1}^{n+k} Z_{1}^{j}= \frac{Z_{1}^{n+1}-Z_{1}^{n+k+1}}{1-Z_{1}}, \\ & \bigl\Vert E_{n+k}(t)-E_{n}(t) \bigr\Vert \leq \sum_{j=n+1}^{n+k} Z_{2}^{j}= \frac{Z_{2}^{n+1}-Z_{2}^{n+k+1}}{1-Z_{2}}, \\ & \bigl\Vert I_{n+k}(t)-I_{n}(t) \bigr\Vert \leq \sum_{j=n+1}^{n+k} Z_{3}^{j}= \frac{Z_{3}^{n+1}-Z_{3}^{n+k+1}}{1-Z_{3}}, \\ & \bigl\Vert Q_{n+k}(t)-Q_{n}(t) \bigr\Vert \leq \sum_{j=n+1}^{n+k} Z_{4}^{j}= \frac{Z_{4}^{n+1}-Z_{4}^{n+k+1}}{1-Z_{4}}, \\ & \bigl\Vert R_{n+k}(t)-R_{n}(t) \bigr\Vert \leq \sum_{i=n+1}^{n+k} Z_{5}^{j}= \frac{Z_{5}^{n+1}-Z_{5}^{n+k+1}}{1-Z_{5}}, \end{aligned} $$

with $Z_{i}=\frac{1-\chi }{B(\chi )} \eta _{i} + \frac{\chi }{B(\chi ) \Gamma (\chi )} \bar{b} \eta _{i}<1$ by hypothesis. Similar to the method as mentioned in [31], we can easily obtain the existence of a unique solution for system (2). □

## Hyers–Ulam stability

### Definition

([31])

The ABC fractional integral system given by Eq. (18) is said to be Hyers–Ulam stable if there exist constants $\Delta _{i} >0$, $i \in \mathbf{N}^{5}$ satisfying: For every $\gamma _{i} >0$, $i \in \mathbf{N}^{5}$, for

31 $$ \begin{aligned} &\biggl\vert S(t)- \frac{1-\chi }{B(\chi )} \mathfrak{K}_{1} \bigl(\chi , t, S(t) \bigr)+ \frac{\chi }{B(\chi ) \Gamma (\chi )} \times \int _{0}^{t}(t- \vartheta )^{\chi -1} \mathfrak{K}_{1} \bigl(\chi , \vartheta , S( \vartheta ) \bigr) \,d\vartheta \biggr\vert \leq \gamma _{1}, \\ &\biggl\vert E(t)- \frac{1-\chi }{B(\chi )} \mathfrak{K}_{2} \bigl( \chi , t, E(t) \bigr)+ \frac{\chi }{B(\chi ) \Gamma (\chi )} \times \int _{0}^{t}(t- \vartheta )^{\chi -1} \mathfrak{K}_{2} \bigl(\chi , \vartheta , E( \vartheta ) \bigr) \,d\vartheta \biggr\vert \leq \gamma _{2} , \\ &\biggl\vert I(t)- \frac{1-\chi }{B(\chi )} \mathfrak{K}_{3} \bigl( \chi , t, I(t) \bigr)+ \frac{\chi }{B(\chi ) \Gamma (\chi )} \times \int _{0}^{t}(t- \vartheta )^{\chi -1} \mathfrak{K}_{3} \bigl(\chi , \vartheta , I( \vartheta ) \bigr) \,d\vartheta \biggr\vert \leq \gamma _{3} , \\ &\biggl\vert Q(t)- \frac{1-\chi }{B(\chi )} \mathfrak{K}_{4} \bigl( \chi , t, Q(t) \bigr)+ \frac{\chi }{B(\chi ) \Gamma (\chi )} \times \int _{0}^{t}(t- \vartheta )^{\chi -1} \mathfrak{K}_{4} \bigl(\chi , \vartheta , Q( \vartheta ) \bigr) \,d\vartheta \biggr\vert \leq \gamma _{4} , \\ &\biggl\vert R(t)- \frac{1-\chi }{B(\chi )} \mathfrak{K}_{5} \bigl( \chi , t, R(t) \bigr)+ \frac{\chi }{B(\chi ) \Gamma (\chi )} \times \int _{0}^{t}(t- \vartheta )^{\chi -1} \mathfrak{K}_{5} \bigl(\chi , \vartheta , R( \vartheta ) \bigr) \,d\vartheta \biggr\vert \leq \gamma _{5}, \end{aligned} $$

there exist $(\dot{S}(t), \dot{E}(t), \dot{I}(t), \dot{Q}(t), \dot{R}(t))$ which satisfy

32 $$ \begin{aligned} &\dot{S}(t)= \frac{1-\chi }{B(\chi )} \mathfrak{K}_{1} \bigl(\chi , t, S(t) \bigr)+ \frac{\chi }{B(\chi ) \Gamma (\chi )} \times \int _{0}^{t}(t- \vartheta )^{\chi -1} \mathfrak{K}_{1} \bigl(\chi , \vartheta , \dot{S}( \vartheta ) \bigr) \,d\vartheta , \\ &\dot{E}(t)= \frac{1-\chi }{B(\chi )} \mathfrak{K}_{2} \bigl(\chi , t, E(t) \bigr)+ \frac{\chi }{B(\chi ) \Gamma (\chi )} \times \int _{0}^{t}(t- \vartheta )^{\chi -1} \mathfrak{K}_{2} \bigl(\chi , \vartheta , \dot{E}( \vartheta ) \bigr) \,d\vartheta , \\ &\dot{I}(t)= \frac{1-\chi }{B(\chi )} \mathfrak{K}_{3} \bigl(\chi , t, I(t) \bigr)+ \frac{\chi }{B(\chi ) \Gamma (\chi )} \times \int _{0}^{t}(t- \vartheta )^{\chi -1} \mathfrak{K}_{3} \bigl(\chi , \vartheta , \dot{I}( \vartheta ) \bigr) \,d\vartheta , \\ &\dot{Q}(t)= \frac{1-\chi }{B(\chi )} \mathfrak{K}_{4} \bigl(\chi , t, Q(t) \bigr)+ \frac{\chi }{B(\chi ) \Gamma (\chi )} \times \int _{0}^{t}(t- \vartheta )^{\chi -1} \mathfrak{K}_{4} \bigl(\chi , \vartheta , \dot{Q}( \vartheta ) \bigr) \,d\vartheta , \\ &\dot{R}(t)= \frac{1-\chi }{B(\chi )} \mathfrak{K}_{5} \bigl(\chi , t, R(t) \bigr)+ \frac{\chi }{B(\chi ) \Gamma (\chi )} \times \int _{0}^{t}(t- \vartheta )^{\chi -1} \mathfrak{K}_{5} \bigl(\chi , \vartheta , \dot{R}( \vartheta ) \bigr) \,d\vartheta , \end{aligned} $$

such that

33 $$ \begin{aligned} &\bigl\vert S(t)-\dot{S}(t) \bigr\vert \leq \zeta _{1} \gamma _{1},\qquad \bigl\vert E(t)- \dot{E}(t) \bigr\vert \leq \zeta _{2} \gamma _{2},\qquad \bigl\vert I(t)- \dot{I}(t) \bigr\vert \leq \zeta _{3} \gamma _{3}, \\ &\bigl\vert Q(t)-\dot{Q}(t) \bigr\vert \leq \zeta _{4} \gamma _{4},\qquad \bigl\vert R(t)-\dot{R}(t) \bigr\vert \leq \zeta _{5} \gamma _{5}. \end{aligned} $$

### Theorem 4

*Model* (2) *is Hyers–Ulam stable subject to the condition* *J*.

### Proof

Thanks to Theorem 3, the proposed ABC fractional model (2) has a unique solution $({S}(t), {E}(t), {I}(t), {Q}(t), {R}(t))$ satisfying (18). Then we have

34 $$\begin{aligned}& \begin{aligned} \bigl\Vert S(t)-\dot{S}(t) \bigr\Vert &\leq \frac{1-\chi }{B(\chi )} \bigl\Vert \mathfrak{K}_{1} \bigl(\chi , t, S(t) \bigr)-\mathfrak{K}_{1} \bigl(\chi , t, \dot{S}(t) \bigr) \bigr\Vert \\ &\quad {}+\frac{\chi }{B(\chi ) \Gamma (\chi )} \int _{0}^{t}(t-\vartheta )^{ \chi -1} \bigl\Vert \mathfrak{K}_{1} \bigl(\chi , t, S(t) \bigr)- \mathfrak{K}_{1} \bigl( \chi , t, \dot{S}(t) \bigr) \bigr\Vert \,d\vartheta \\ &\leq \biggl[\frac{1-\chi }{B(\chi )}+ \frac{\chi }{B(\chi ) \Gamma (\chi )} \biggr] \chi _{1} \Vert S-\dot{S} \Vert , \end{aligned} \end{aligned}$$

35 $$\begin{aligned}& \begin{aligned} \bigl\Vert E(t)-\dot{E}(t) \bigr\Vert &\leq \frac{1-\chi }{B(\chi )} \bigl\Vert \mathfrak{K}_{2} \bigl(\chi , t, E(t) \bigr)-\mathfrak{K}_{2} \bigl(\chi , t, \dot{E}(t) \bigr) \bigr\Vert \\ &\quad {}+\frac{\chi }{B(\chi ) \Gamma (\chi )} \int _{0}^{t}(t-\vartheta )^{ \chi -1} \bigl\Vert \mathfrak{K}_{2} \bigl(\chi , t, E(t) \bigr)- \mathfrak{K}_{2} \bigl( \chi , t, \dot{E}(t) \bigr) \bigr\Vert \,d\vartheta \\ &\leq \biggl[\frac{1-\chi }{B(\chi )}+ \frac{\chi }{B(\chi ) \Gamma (\chi )} \biggr] \chi _{2} \Vert E-\dot{E} \Vert , \end{aligned} \end{aligned}$$

36 $$\begin{aligned}& \begin{aligned} \bigl\Vert I(t)-\dot{I}(t) \bigr\Vert &\leq \frac{1-\chi }{B(\chi )} \bigl\Vert \mathfrak{K}_{3} \bigl(\chi , t, I(t) \bigr)-\mathfrak{K}_{3} \bigl(\chi , t, \dot{I}(t) \bigr) \bigr\Vert \\ &\quad {}+\frac{\chi }{B(\chi ) \Gamma (\chi )} \int _{0}^{t}(t-\vartheta )^{ \chi -1} \bigl\Vert \mathfrak{K}_{3} \bigl(\chi , t, I(t) \bigr)- \mathfrak{K}_{3} \bigl( \chi , t, \dot{I}(t) \bigr) \bigr\Vert \,d\vartheta \\ &\leq \biggl[\frac{1-\chi }{B(\chi )}+ \frac{\chi }{B(\chi ) \Gamma (\chi )} \biggr] \chi _{3} \Vert I-\dot{I} \Vert , \end{aligned} \end{aligned}$$

37 $$\begin{aligned}& \begin{aligned} \bigl\Vert Q(t)-\dot{Q}(t) \bigr\Vert &\leq \frac{1-\chi }{B(\chi )} \bigl\Vert \mathfrak{K}_{4} \bigl(\chi , t, Q(t) \bigr)-\mathfrak{K}_{4} \bigl(\chi , t, \dot{Q}(t) \bigr) \bigr\Vert \\ &\quad {}+\frac{\chi }{B(\chi ) \Gamma (\chi )} \int _{0}^{t}(t-\vartheta )^{ \chi -1} \bigl\Vert \mathfrak{K}_{4} \bigl(\chi , t, Q(t) \bigr)- \mathfrak{K}_{4} \bigl( \chi , t, \dot{Q}(t) \bigr) \bigr\Vert \,d\vartheta \\ &\leq \biggl[\frac{1-\chi }{B(\chi )}+ \frac{\chi }{B(\chi ) \Gamma (\chi )} \biggr] \chi _{4} \Vert Q-\dot{Q} \Vert , \end{aligned} \end{aligned}$$

38 $$\begin{aligned}& \begin{aligned} \bigl\Vert R(t)-\dot{R_{h}}(t) \bigr\Vert &\leq \frac{1-\chi }{B(\chi )} \bigl\Vert \mathfrak{K}_{5} \bigl(\chi , t, R(t) \bigr)-\mathfrak{K}_{5} \bigl(\chi , t, \dot{R}(t) \bigr) \bigr\Vert \\ &\quad {}+\frac{\chi }{B(\chi ) \Gamma (\chi )} \int _{0}^{t}(t-\vartheta )^{ \chi -1} \bigl\Vert \mathfrak{K}_{5} \bigl(\chi , t, R(t) \bigr)- \mathfrak{K}_{5} \bigl( \chi , t, \dot{R}(t) \bigr) \bigr\Vert \,d\vartheta \\ &\leq \biggl[\frac{1-\chi }{B(\chi )}+ \frac{\chi }{B(\chi ) \Gamma (\chi )} \biggr] \chi _{5} \Vert R-\dot{R} \Vert . \end{aligned} \end{aligned}$$

Taking $\gamma _{i} = \chi _{i}$, $\Delta _{i} = \frac{1-\chi }{B(\chi )}+ \frac{\chi }{B(\chi ) \Gamma (\chi )}$ implies

39 $$ \bigl\Vert S(t)-\dot{S}(t) \bigr\Vert \leq \gamma _{1} \Delta _{1}. $$

Similarly,

40 $$ \textstyle\begin{cases} \Vert E(t)-\dot{E}(t) \Vert \leq \gamma _{2} \Delta _{2}, \\ \Vert I(t)-\dot{I}(t) \Vert \leq \gamma _{3} \Delta _{3}, \\ \Vert Q(t)-\dot{Q}(t) \Vert \leq \gamma _{4} \Delta _{4}, \\ \Vert R(t)-\dot{R}(t) \Vert \leq \gamma _{5} \Delta _{5}. \end{cases} $$

System (18) is Hyers–Ulam stable by taking into account (39) and (40), hence model (2) is Hyers–Ulam stable. □

## Numerical scheme

To solve our proposed model we incorporate the Toufik–Atangana scheme [32]. For this we consider the first equation of (2). We have

41 $$ ^{ABC}_{0}\mathcal{D}_{t}^{\chi }S(t)=G_{1} \bigl(t,S(t) \bigr), \qquad S(0)=S_{0}, $$

the solution of which is

42 $$ S(t)=S(0)+\frac{1-\chi }{F(\chi )}G_{1} \bigl(t,S(t) \bigr)+ \frac{\chi }{F(\chi )\Gamma (\chi )} \int _{0}^{t}G_{1} \bigl(k,S(k) \bigr) (t-k)^{ \chi -1}\,dk. $$

Applying Lagrange’s interpolation polynomial on the interval $[t_{k},t_{k+1}]$ to the equality $G_{1}(y,S(y))=\frac{\Lambda }{\alpha _{3}}-b_{0} \frac{(I(y)+A(y)S(y))}{k(N)}-S(y)$ leads to

43 $$ \begin{aligned} S_{k} &\approx \frac{1}{h} \bigl[(y-t_{k-1})G_{1} \bigl(t_{k},S(t_{k}),I(t_{k}),A(t_{k}) \bigr) \\ &\quad {}-(y-t_{k})G_{1} \bigl(t_{k+1},S(t_{k+1}),I(t_{k+1}),A(t_{k+1}) \bigr) \bigr], \end{aligned} $$

where $h=t_{k}-t_{k-1}$. Now, substituting (43) into (42), we have

44 $$ \begin{aligned} &S(t_{n+1}) \\ &\quad =S(0)+ \frac{1-\chi }{F(\chi )}G_{1} \bigl(t_{k},S(t_{k}),E(t_{k}),I(t_{k}),Q(t_{k}),R(t_{k}) \bigr) \\ &\qquad {}+\frac{\chi }{F(\chi )\Gamma (\chi )} \\ &\qquad {}\times\sum_{j=1}^{n} \left(\textstyle\begin{array}{l} \frac{G_{1}(t_{j},S(t_{j}),E(t_{j}),I(t_{j}),Q(t_{j}),R(t_{j}))}{h} \int _{t_{j}}^{t_{j+1}}(y-t_{j-1})(t_{n+1}-y)^{\chi -1}\,dy \\ \quad {}- \frac{G_{1}(t_{j-1},S(t_{j-1}),E(t_{j-1}),I(t_{j-1}),Q(t_{j-1}),R(t_{j-1}))}{h} \int _{t_{j}}^{t_{j+1}}(y-t_{j-1})(t_{n+1}-y)^{\chi -1}\,dy \end{array}\displaystyle \right) \\ &\qquad{}\times S(0)+\frac{1+\chi }{F(\chi )}G_{1} \bigl(t_{n},S(t_{n}),E(t_{n}),I(t_{n}),Q(t_{n}),R(t_{n}) \bigr) \\ &\qquad {}+\frac{\chi }{F(\chi )\Gamma (\chi )}\sum_{j=1}^{n} \left(\textstyle\begin{array}{l} \frac{G_{1}(t_{j},S(t_{j}),E(t_{j}),I(t_{j}),Q(t_{j}),R(t_{j}))}{h} \Upsilon _{j-1} \\ - \frac{G_{1}(t_{j-1},S(t_{j-1}),E(t_{j-1}),I(t_{j-1}),Q(t_{j-1}),R(t_{j-1}))}{h} \Upsilon _{j} \end{array}\displaystyle \right), \end{aligned} $$

where

45 $$\begin{aligned}& \begin{aligned} \Upsilon _{j-1} &= \int _{t_{j}}^{t_{j+1}}(y-t_{j-1}) (t_{n+1}-y)^{ \chi -1}\,dy \\ &= -\frac{1}{\chi } \bigl[ (t_{j+1}-t_{j-1}) (t_{n+1}-t_{j+1})^{\chi } - (t_{j}-t_{j-1}) (t_{n+1}-t_{j})^{ \chi } \bigr] \\ &\quad {}-\frac{1}{\chi (\chi +1)} \bigl[ (t_{n+1}-t_{j+1})^{\chi +1}(t_{n+1}-t_{j+1})^{ \chi } - (t_{n+1}-t_{j})^{\chi +1} \bigr], \end{aligned} \end{aligned}$$

46 $$\begin{aligned}& \begin{aligned} \Upsilon _{j} &= \int _{t_{j}}^{t_{j+1}}(y-t_{j-1}) (t_{n+1}-y)^{\chi -1}\,dy \\ &= -\frac{1}{\chi } \bigl[ (t_{j+1}-t_{j-1}) (t_{n+1}-t_{j+1})^{\chi } \bigr] \\ &\quad {}-\frac{1}{\chi (\chi +1)} \bigl[(t_{n+1}-t_{j+1})^{\chi +1}(t_{n+1}-t_{j})^{ \chi +1} \bigr]. \end{aligned} \end{aligned}$$

Incorporating $t_{j}=jh$ into (45) and (46) leads to

47 $$\begin{aligned}& \Upsilon _{j-1}= \frac{h^{\chi +1}}{\chi (\chi +1)} \bigl[(n+1-j)^{\chi }(n-j+2+ \chi )-(n-j)^{\chi }(n-j+2+2\chi ) \bigr] , \end{aligned}$$

48 $$\begin{aligned}& \Upsilon _{j}= \frac{h^{\chi +1}}{\chi (\chi +1)} \bigl[(n+1-j)^{\chi +1}-(n-j)^{ \chi }(n-j+1+ \chi ) \bigr]. \end{aligned}$$

Equation (44) with the help of (47) and (48) becomes

49 $$ \begin{aligned} S(t_{n+1}) &= S(t_{0})+\frac{1-\chi }{F(\chi )}G_{1} \bigl(t_{n},S(t_{n}),E(t_{n}),I(t_{n}),Q(t_{n}),R(t_{n}) \bigr) \\ &\quad {}+ \frac{\chi }{F(\chi )\Gamma (\chi )} \\ &\quad {}\times\sum_{j=1}^{n} \left(\textstyle\begin{array}{l} \frac{G_{1}(t_{j},S(t_{j}),E(t_{j}),I(t_{j}),Q(t_{j}),R(t_{j}))}{\Gamma (\chi +2)} \\ \quad {}\times h^{\chi }[(n+1-j)^{\chi }(n-j+2+\chi )-(n-j)^{\chi }(n-j+2+2\chi )] \\ \quad {}- \frac{G_{1}(t_{j-1},S(t_{j-1}),E(t_{j-1}),I(t_{j-1}),Q(t_{j-1}),R(t_{j-1}))}{\Gamma (\chi +2)} \\ \quad {}\times h^{\chi }[(n+1-j)^{\chi +1}-(n-j)^{\chi }(n-j+1+\chi )] \end{array}\displaystyle \right). \end{aligned} $$

Similarly,

50 $$\begin{aligned}& \begin{aligned} E(t_{n+1}) &= E(t_{0})+ \frac{1-\chi }{F(\chi )}G_{1} \bigl(t_{n},S(t_{n}),E(t_{n}),I(t_{n}),Q(t_{n}),R(t_{n}) \bigr) \\ &\quad {}+ \frac{\chi }{F(\chi )\Gamma (\chi )} \\ &\quad {}\times\sum_{j=1}^{n} \left(\textstyle\begin{array}{l} \frac{G_{1}(t_{j},S(t_{j}),E(t_{j}),I(t_{j}),Q(t_{j}),R(t_{j}))}{\Gamma (\chi +2)} \\ \quad {}\times h^{\chi }[(n+1-j)^{\chi }(n-j+2+\chi )-(n-j)^{\chi }(n-j+2+2\chi )] \\ \quad {}- \frac{G_{1}(t_{j-1},S(t_{j-1}),E(t_{j-1}),I(t_{j-1}),Q(t_{j-1}),R(t_{j-1}))}{\Gamma (\chi +2)} \\ \quad {}\times h^{\chi }[(n+1-j)^{\chi +1}-(n-j)^{\chi }(n-j+1+\chi )] \end{array}\displaystyle \right), \end{aligned} \end{aligned}$$

51 $$\begin{aligned}& \begin{aligned} I(t_{n+1}) &= I(t_{0})+ \frac{1-\chi }{F(\chi )}G_{1} \bigl(t_{n},S(t_{n}),E(t_{n}),I(t_{n}),Q(t_{n}),R(t_{n}) \bigr) \\ &\quad {}+ \frac{\chi }{F(\chi )\Gamma (\chi )} \\ &\quad {}\times\sum_{j=1}^{n} \left(\textstyle\begin{array}{l} \frac{G_{1}(t_{j},S(t_{j}),E(t_{j}),I(t_{j}),Q(t_{j}),R(t_{j}))}{\Gamma (\chi +2)} \\ \quad {}\times h^{\chi }[(n+1-j)^{\chi }(n-j+2+\chi )-(n-j)^{\chi }(n-j+2+2\chi )] \\ \quad {}- \frac{G_{1}(t_{j-1},S(t_{j-1}),E(t_{j-1}),I(t_{j-1}),Q(t_{j-1}),R(t_{j-1}))}{\Gamma (\chi +2)} \\ \quad {}\times h^{\chi }[(n+1-j)^{\chi +1}-(n-j)^{\chi }(n-j+1+\chi )] \end{array}\displaystyle \right), \end{aligned} \end{aligned}$$

52 $$\begin{aligned}& \begin{aligned} Q(t_{n+1}) &= Q(t_{0})+ \frac{1-\chi }{F(\chi )}G_{1} \bigl(t_{n},S(t_{n}),E(t_{n}),I(t_{n}),Q(t_{n}),R(t_{n}) \bigr) \\ &\quad {}+ \frac{\chi }{F(\chi )\Gamma (\chi )} \\ &\quad {}\times\sum_{j=1}^{n} \left(\textstyle\begin{array}{l} \frac{G_{1}(t_{j},S(t_{j}),E(t_{j}),I(t_{j}),Q(t_{j}),R(t_{j}))}{\Gamma (\chi +2)} \\ \quad {}\times h^{\chi }[(n+1-j)^{\chi }(n-j+2+\chi )-(n-j)^{\chi }(n-j+2+2\chi )] \\ \quad {}- \frac{G_{1}(t_{j-1},S(t_{j-1}),E(t_{j-1}),I(t_{j-1}),Q(t_{j-1}),R(t_{j-1}))}{\Gamma (\chi +2)} \\ \quad {}\times h^{\chi }[(n+1-j)^{\chi +1}-(n-j)^{\chi }(n-j+1+\chi )] \end{array}\displaystyle \right), \end{aligned} \end{aligned}$$

53 $$\begin{aligned}& \begin{aligned} R(t_{n+1}) &= R(t_{0})+\frac{1-\chi }{F(\chi )}G_{1} \bigl(t_{n},S(t_{n}),E(t_{n}),I(t_{n}),Q(t_{n}),R(t_{n}) \bigr) \\ &\quad {}+ \frac{\chi }{F(\chi )\Gamma (\chi )} \\ &\quad {}\times\sum_{j=1}^{n} \left(\textstyle\begin{array}{l} \frac{G_{1}(t_{j},S(t_{j}),E(t_{j}),I(t_{j}),Q(t_{j}),R(t_{j}))}{\Gamma (\chi +2)} \\ \quad {}\times h^{\chi }[(n+1-j)^{\chi }(n-j+2+\chi )-(n-j)^{\chi }(n-j+2+2\chi )] \\ \quad {}- \frac{G_{1}(t_{j-1},S(t_{j-1}),E(t_{j-1}),I(t_{j-1}),Q(t_{j-1}),R(t_{j-1}))}{\Gamma (\chi +2)} \\ \quad {}\times h^{\chi }[(n+1-j)^{\chi +1}-(n-j)^{\chi }(n-j+1+\chi )] \end{array}\displaystyle \right). \end{aligned} \end{aligned}$$

## Graphical results

**Figure 1:**
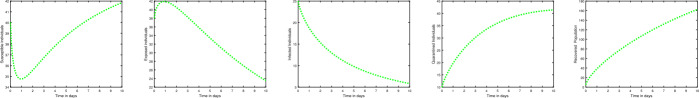
For $\chi =0.8$, plots of *S*, *E*, *I*, *Q*, *R*

**Figure 2:**
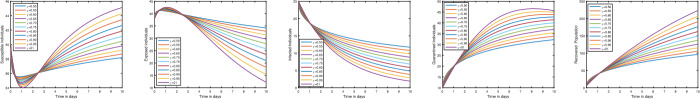
Profiles for the first set of initial conditions at different *χ* values i.e. $\chi =0.50, 0.55, 0.60, 0.65, 0.70, 0.75, 0.80, 0.85, 0.90$, and $\alpha =0.2$

**Figure 3:**
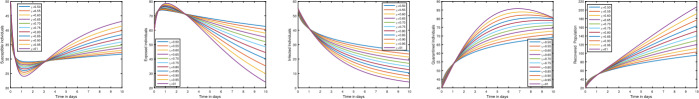
Profiles for the second set of initial conditions at different *χ* values i.e. $\chi =0.50, 0.55, 0.60, 0.65, 0.70, 0.75, 0.80, 0.85, 0.90$, and $\alpha =0.2$

**Figure 4:**
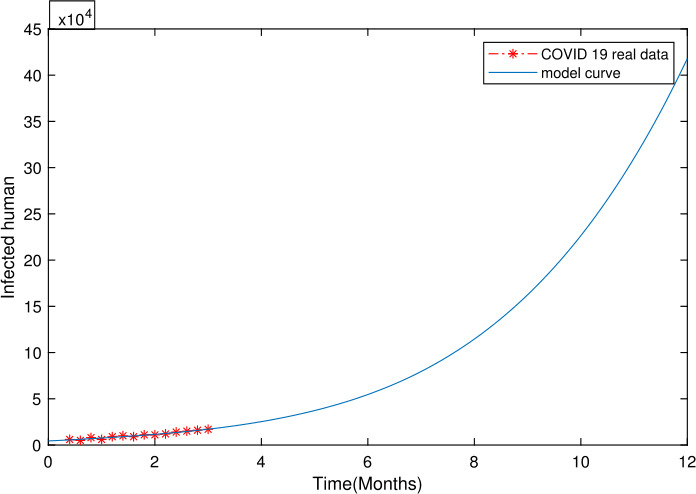
The incidence data of COVID-19 from Khyber Pakhtunkhwa, Pakistan

We get the numerical simulations based on (49)–(53) with parameter values given in Table 1. Figure 1 represents the dynamics of all the five population classes i.e. *S*, *E*, *I*, *Q*, and *R* when $\chi =0.8$. Figure 2 represents the numerical simulation results for $\alpha =0.2$ based on the Mittag—Leffler generalized function which is characterized by the crossover property when stretched from one operator to another. The operator has a statistical representation making it more viable. In Fig. 2, the susceptible human population increases as the fractional order *χ* derivative increases. In Fig. 2 the number of the exposed decreases as the fractional order *χ* value increases. Figure 2 depicts COVID-19 infected people, the number of which decreases as the fractional order derivative increases. Similarly, Fig. 2 depicts quarantined people and the number of quarantined people increases as the fractional order derivative increases. In Fig. 2 the recovered human population increases as the fractional order values increase. Figure 3 represents the numerical simulation results for $\alpha =0.3$ and for different values of fractional parameter *χ*. Figure 4. shows the real data plot against the model infected people class.

**Table 1:** Values and descriptions of the parameters (based on data [26])

Symbols	Values	References
*b̄*	0.028	[26]
*ᾱ*	0.2	[26]
*μ̄*	0.011	[26]
$\bar{d_{1}}$	0.2	[26]
$\bar{d_{2}}$	0.08	[26]
$\bar{d_{3}}$	0.06	[26]
*λ̄*	0.3	[26]
*β̄*	0.5	[26]
*γ̄*	0.3	[26]
*η̄*	0.6	[26]
*τ̄*	0.1	[26]
*ϵ̄*	0.3	[26]

## Optimal control analysis

We use two control variables i.e. social distancing $u_{1}(t)$ and treatment $u_{2}(t)$ [33–35]. The objective functional is

54 $$ J(u_{1}, u_{2})=\min \int _{0}^{T} \biggl[\mathcal{G}_{1}S(t)+ \mathcal{G}_{2}E(t)+ \mathcal{G}_{3}I(t)+ \frac{1}{2} \bigl(Z_{1}u_{1}^{2}(t)+Z_{2}u_{2}^{2}(t) \bigr) \biggr]\,dt. $$

Subject to the state system, model (2) is modified to (55) after incorporating the control variable

55 $$ \textstyle\begin{cases} {}^{\mathrm{ABC}} \mathbb{D}_{0, t}^{\chi } [S(t) ]= \bar{b}- \bar{\beta } S(t)I(t) (1+\bar{\delta } I(t) ) - (\bar{\eta } + \bar{\mu } + \bar{d}_{3}+u_{1}(t) )S(t), \\ {}^{\mathrm{ABC}} \mathbb{D}_{0, t}^{\chi } [E(t) ]= \bar{\beta } S(t) I(t) (1+\bar{\delta } I(t) ) - (\bar{\lambda } + \bar{\mu } + \bar{d}_{2}+u_{1}(t) )E(t), \\ {}^{\mathrm{ABC}} \mathbb{D}_{0, t}^{\chi } [I(t) ] = \bar{\lambda } E(t)-( \bar{\mu } +\bar{\epsilon }+\bar{\gamma } + \bar{d}_{1})I(t)- (u_{1}(t)+u_{2}(t) ) I(t), \\ {}^{\mathrm{ABC}} \mathbb{D}_{0, t}^{\chi } [Q(t) ] =\bar{d}_{3}S(t)+ \bar{d}_{2}E(t)+\bar{d}_{1}I(t) +u_{2}(t) I(t) - (\bar{\mu } + \bar{\tau }^{\chi }+u_{1}(t) )Q(t), \\ {}^{\mathrm{ABC}} \mathbb{D}_{0, t}^{\chi } [R(t) ] = \bar{\eta } S(t)+ \bar{\tau }^{\chi }Q(t)+\bar{\gamma } I(t) +u_{1}(t)S(t)+u_{1}(t)E(t) \\ \hphantom{{}^{\mathrm{ABC}} \mathbb{D}_{0, t}^{\chi } [R(t) ] ={}}{} +u_{1}(t)I(t) +u_{1}(t)Q(t)-\bar{\mu } R(t) \end{cases} $$

with ICs

56 $$ S(0)\geq 0, \qquad E(0)\geq 0, \qquad I(0)\geq 0, \qquad Q(0)\geq 0, \qquad R(0) \geq 0. $$

In the objective functional (54), $\mathcal{G}_{1}$, $\mathcal{G}_{2}$, and $\mathcal{G}_{3}$ are the relative weights and $Z_{1}$ and $Z_{2}$ measure the associated cost on social distancing and treatment, respectively. Our goal is to find the control function such that

57 $$ J \bigl(u_{1}^{*},u_{2}^{*} \bigr)=\min \bigl\{ J(u_{1},u_{2}),u_{1},u_{2} \in U \bigr\} $$

subject to system (55), where the control set is defined as

58 $$ U= \bigl\{ (u_{1},u_{2})/u_{i}(t) 0\leq u_{i}(t)\leq 1,i=1,2 \bigr\} . $$

The conditions that an optimal solution must satisfy are obtained by using Pontryagin’s maximum principle. This principle translates Eqs. (54)–(55) into a problem characterized with minimizing the following Hamiltonian *H* with regard to control variables:

59 $$ \begin{aligned} H={}&\mathcal{G}_{1}S(t)+ \mathcal{G}_{2}E(t)+\mathcal{G}_{3}I(t)+ \frac{1}{2} \bigl(Z_{1}u_{1}^{2}+Z_{2}u_{2}^{2} \bigr) \\ &{}+\lambda _{1} \bigl[\bar{b}- \bar{\beta } S(t)I(t) \bigl(1+\bar{ \delta } I(t) \bigr) - \bigl( \bar{\eta } + \bar{\mu } + \bar{d}_{3}+u_{1}(t) \bigr)S(t) \bigr] \\ &{}+\lambda _{2} \bigl[\bar{\beta } S(t) I(t) \bigl(1+\bar{\delta } I(t) \bigr) - \bigl( \bar{\lambda } + \bar{\mu } + \bar{d}_{2}+u_{1}(t) \bigr)E(t) \bigr] \\ &{}+\lambda _{3} \bigl[\bar{\lambda } E(t)-(\bar{\mu } +\bar{\epsilon }+ \bar{\gamma } +\bar{d}_{1})I(t)- \bigl(u_{1}(t)+u_{2}(t) \bigr) I(t) \bigr] \\ &{}+\lambda _{4} \bigl[\bar{d}_{3}S(t)+ \bar{d}_{2}E(t)+\bar{d}_{1}I(t) +u_{2}(t) I(t) - \bigl(\bar{\mu } +\bar{\tau }^{\chi }+u_{1}(t) \bigr)Q(t) \bigr] \\ &{}+\lambda _{5} \bigl[\bar{\eta } S(t)+\bar{\tau }^{\chi }Q(t)+\bar{\gamma } I(t) +u_{1}(t)S(t) +u_{1}(t)E(t) \\ &{}+u_{1}(t)I(t) +u_{1}(t)Q(t)- \bar{\mu } R(t) \bigr], \end{aligned} $$

where $\lambda _{1}(t)$, $\lambda _{2}(t)$, $\lambda _{3}(t)$, $\lambda _{4}(t)$, and $\lambda _{5}(t)$ are made up of the adjoint variables. The system solution is determined by taking the partial derivatives of Hamiltonian (59) with respect to the associated state variable.

Following [32], we obtain the necessary optimality conditions for the system of equations (54) and (55):

60 $$\begin{aligned}& \left . \textstyle\begin{array}{l} ^{\mathrm{ABC}} \mathbb{D}_{0, t}^{\chi }[S(t)]= \frac{\partial H}{\partial \lambda _{S}}(t),\qquad {}^{\mathrm{ABC}} \mathbb{D}_{0, t}^{\chi }[E(t)]= \frac{\partial H}{\partial \lambda _{E}}(t) , \\ ^{\mathrm{ABC}} \mathbb{D}_{0, t}^{\chi }[I(t)]= \frac{\partial H}{\partial \lambda _{A}}(t),\qquad {}^{\mathrm{ABC}} \mathbb{D}_{0, t}^{\chi }[Q(t)]= \frac{\partial H}{\partial \lambda _{I}}(t), \\ ^{\mathrm{ABC}} \mathbb{D}_{0, t}^{\chi }[R(t)]= \frac{\partial H}{\partial \lambda _{R}}(t), \end{array}\displaystyle \right \} \end{aligned}$$

61 $$\begin{aligned}& \left . \textstyle\begin{array}{l} {}_{t}^{\mathrm{ABC}} \mathbb{D}_{T}^{\chi } \lambda _{S}(t)=- \frac{\partial H}{\partial S}(t), \qquad {}_{t}^{\mathrm{ABC}} \mathbb{D}_{T}^{\chi } \lambda _{E}(t)=-\frac{\partial H}{\partial E}(t) , \\ {}_{t}^{\mathrm{ABC}} \mathbb{D}_{T}^{\chi } \lambda _{I}(t)=- \frac{\partial H}{\partial I}(t), \qquad {}_{t}^{\mathrm{ABC}} \mathbb{D}_{T}^{\chi } \lambda _{Q}(t)=-\frac{\partial H}{\partial Q}(t) , \\ {}_{t}^{\mathrm{ABC}} \mathbb{D}_{T}^{\chi } \lambda _{R}(t)=- \frac{\partial H}{\partial R}(t), \qquad \frac{\partial H}{\partial u}(t)=0. \end{array}\displaystyle \right \} \end{aligned}$$

### Theorem 5

*In view of the optimal controls*, $(u_{1}^{*},u_{2}^{*})$*is the solution of the above control system* (54)*–*(55), *then we can find the adjoint variables* $\lambda _{i}(t)$ *for* $i=S,E,I,Q,R$, *satisfying*

62 $$ {}_{t}^{\mathrm{ABC}} \mathbb{D}_{T}^{\chi } \lambda _{i}(t)= \frac{\partial H}{\partial i}, $$

*where* $i=S,E,I,Q,R$ *and with the transversality conditions*

63 $$ \lambda _{i}(T)=0 \quad \textit{for } i=S,E,I,Q,R. $$

*Furthermore*, *the optimal control variables* $u_{1}^{*}(t)$, $u_{2}^{*}(t)$ *are defined by*

64 $$\begin{aligned}& u^{*}_{1}(t) = \max \biggl\{ \min \biggl\{ \frac{ \mathcal{X}}{Z_{1}},1 \biggr\} ,0 \biggr\} , \\& u^{*}_{2}(t) = \max \biggl\{ \min \biggl\{ \frac{(\lambda _{I}(t)-\lambda _{Q}(t))I^{*}(t)}{Z_{2}},1 \biggr\} ,0 \biggr\} , \end{aligned}$$

*where*

$$\begin{aligned} \mathcal{X} =& \bigl(\lambda _{1}(t)+\lambda _{R}(t) \bigr)S^{*}(t)+ \bigl(\lambda _{E}(t)+ \lambda _{R}(t) \bigr)E^{*}(t) \\ &{}+ \bigl(\lambda _{R}(t)-\lambda _{I}(t) \bigr)I^{*}(t)+ \bigl( \lambda _{Q}(t)+\lambda _{R}(t) \bigr)Q^{*}(t). \end{aligned}$$

### Proof

Using (62), we reach the adjoint system

65 $$\begin{aligned} &\begin{aligned} {}_{t}^{\mathrm{ABC}} \mathbb{D}_{T}^{\chi } \lambda _{S}(t)={}& {-} \mathcal{G}_{1}+ \lambda _{S}(t) \bigl[\beta I(1+\delta I)+\eta +\mu +d_{3}+\mu _{1}(t) \bigr], \\ &{} - \lambda _{E}(t)\beta I(1+\delta I)-\lambda _{Q}(t)d_{3}- \lambda _{R}(t) \bigl[\eta +\mu _{1}(t) \bigr], \end{aligned} \\ &{}_{t}^{\mathrm{ABC}} \mathbb{D}_{T}^{\chi } \lambda _{E}(t)= - \mathcal{G}_{2}+ \lambda _{E}(t) \bigl[\lambda +\mu +d_{2}+\mu _{1}(t) \bigr] -\lambda _{I}(t) \lambda -\lambda _{Q}(t) d_{3}-\lambda _{5}(t)\mu _{1}(t), \\ &\begin{aligned} {}_{t}^{\mathrm{ABC}} \mathbb{D}_{T}^{\chi } \lambda _{I}(t)={}& {-} \mathcal{G}_{3}+ \lambda _{S}(t) \bigl[\beta S(1+2\delta I) \bigr]-\lambda _{E}(t) \bigl[ \beta S(1+2\delta I) \bigr] \\ &{} + \lambda _{I}(t) \bigl[\mu\, +\in +\gamma +d_{1}+\mu _{1}(t)+\mu _{2}(t) \bigr] \\ &{} -\lambda _{Q}(t) \bigl[d_{1}+\mu _{2}(t) \bigr]- \lambda _{R}(t) \bigl[\gamma +\mu _{1}(t) \bigr], \end{aligned} \\ &{}_{t}^{\mathrm{ABC}} \mathbb{D}_{T}^{\chi } \lambda _{Q}(t)= + \lambda _{Q}(t) \bigl[\mu +\bar{\tau }+\mu _{1}(t) \bigr] -\lambda _{R}(t) \bigl[\bar{\tau }+\mu _{1}(t) \bigr], \\ &{}_{t}^{\mathrm{ABC}} \mathbb{D}_{T}^{\chi } \lambda _{R}(t)= + \lambda _{R}(t)\mu . \end{aligned}$$

Also, by applying $\frac{\partial H}{\partial u i}=0$, we get (64) for $i=1,2$. □

### Scheme for FOCP

For a general initial value problem [36]

66 $$\begin{aligned}& {}^{{AB}}_{C}D^{\chi }_{0,t}e(t) = r \bigl(t,e(t) \bigr), \\& e(0) = e_{0}. \end{aligned}$$

With the help of the fundamental theorem of fractional calculus to Eq. (66), we have

67 $$ e(t)-e(0)=\frac{1-\chi }{\mathfrak{B}(\chi )}r \bigl(t,e(t) \bigr)+ \frac{\chi }{\mathfrak{B}(\chi )\Gamma (\chi )} \int _{0}^{t} r \bigl(\delta ,e( \delta ) \bigr) (t-\delta )^{\chi -1}\,d\delta . $$

With the normalization function $\mathfrak{B}(\chi )=1-\chi +\frac{\chi }{\Gamma (\chi )}$ at $t_{n + 1}$, after discretization, we have

68 $$ \begin{aligned} e_{n+1}(t)-e(0)={}& \frac{\Gamma (\chi )(1-\chi )r(t_{n},e_{n})}{\Gamma (\chi )(1-\chi )+\chi } \\ &{}+ \frac{\chi }{\Gamma (\chi )(1-\chi )+\chi }\sum ^{n}_{m=0} \int _{t_{m}}^{t_{m+1}} r.(t_{n+1}-\delta )^{\chi -1}\,d\delta . \end{aligned} $$

Now, approximating $r(\eta , e(\eta ))$ by the two-step Lagrange interpolation [37], we have

69 $$ r \bigl(\delta , e(\delta ) \bigr)\cong \frac{r(t_{m}, e_{m})(\delta -t_{m-1})}{h}- \frac{r(t_{m-1}, e_{m-1})(\delta -t_{m})}{h}. $$

Now, we get

70 $$ \begin{aligned} &e_{n+1}(t)-e(0) \\ &\quad =\frac{\Gamma (\chi )(1-\chi )}{\Gamma (\chi )(1-\chi )+\chi }r(t_{n},e_{n}) +\frac{1}{(1+\chi )(\Gamma (\chi )(1-\chi )+\chi )}\sum^{n}_{m=0} \\ &\qquad {}\times \bigl\{ h^{\chi }r(t_{m},e_{m}) \bigl\{ (n+1-m)^{\chi }(n-m+2+\chi )-(n-m)^{\chi }(n-m+2+2 \chi ) \bigr\} \\ &\qquad {} -h^{\chi }r(t_{m-1},e_{m-1}) \bigl\{ (n+1-m)^{\chi +1} -(n-m)^{\chi }(n-m+1+ \chi ) \bigr\} \bigr\} . \end{aligned} $$

To get high stability, we incorporate a simple modification [36] such that replacing *h* (step size) with $\chi (h)$ with $\chi (h)=h+O(h^{2}); 0<\chi (h)\leq 1$. This new scheme is a nonstandard one characterized by unconditional stability, and details can be established in [38], and we obtain the following scheme:

71 $$ \begin{aligned} &e_{n+1}(t)-e(0) \\ &\quad =\frac{\Gamma (\chi )(1-\chi )}{\Gamma (\chi )(1-\chi )+\chi }r(t_{n},e_{n}) +\frac{1}{(1+\chi )(\Gamma (\chi )(1-\chi )+\chi )}\sum^{n}_{m=0} \\ &\qquad {}\times \bigl\{ \chi (h)^{\chi }r(t_{m},e_{m}) \bigl\{ (n+1-m)^{\chi }(n-m+2+ \chi )-(n-m)^{\chi }(n-m+2+2 \chi ) \bigr\} \\ &\qquad {} -\chi (h)^{\chi }r(t_{m-1},e_{m-1}) \bigl\{ (n+1-m)^{\chi +1} -(n-m)^{\chi }(n-m+1+ \chi ) \bigr\} \bigr\} . \end{aligned} $$

**Figure 5:**
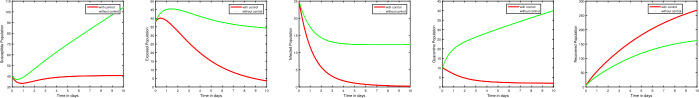
Profiles for behavior of each state variable for the fractional model with and without control

**Figure 6:**
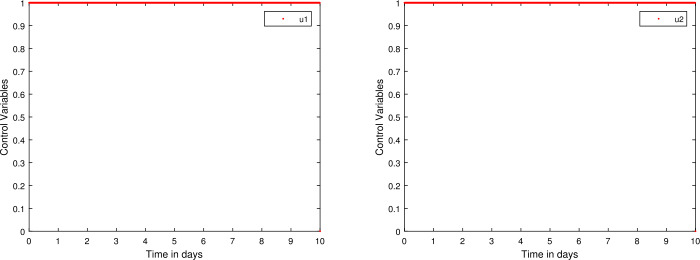
Profiles for the behavior of control variables $u_{1}$ and $u_{2}$

The new scheme is therefore utilized in Eq. (71) to obtain a numerical solution to the state system. Further, we make of use the implicit finite difference method in order to derive the solution of the co-state system Eqs. (55) together with the transversality conditions in Eq. (63). Figure 5 shows the difference between with and without control of each class of the model while Fig. 6 shows the profiles of each control variable.

## Conclusion

In the current analysis, the COVID-19 model has been examined by one of the robust nonlocal fractional operators called the ABC operator in the Caputo sense. COVID-19 is one of the most quickly killing virus. The toxic effects of the infectious disease COVID-19 are very slow-acting and death or life from overdose typically occurs. It is of vehement importance to analyze more critically the dynamic of this subtle virus. The fractional operator employed has been shown to be ideally suitable for studying the transmission dynamics of a disease in the literature. The fractionalized order is *χ*, and consideration was given to the dimensional consistency between the rest of the parameters. As a result, several important features of the proposed fractional version of the model have been documented, such as the model formation, the existence and uniqueness of the solution through the fixed point theorem, invariant region, stability analysis, and, most importantly, the basic number of reproductions. It should be noted that the fractional type disease model under investigation comprehends the behavior of the disease more correctly than the variant of the integer order. In addition, different numerical simulations were carried out by means of an efficient numerical scheme in order to shed more light on the features of the model.

## Data Availability

Not applicable.
